# *QuickStats:* Prevalence* of Untreated Dental Caries^†^ in Primary Teeth^§^ Among Children Aged 2–8 Years, by Age Group and Race/Hispanic Origin — National Health and Nutrition Examination Survey, 2011–2014

**DOI:** 10.15585/mmwr.mm6609a5

**Published:** 2017-03-10

**Figure:**
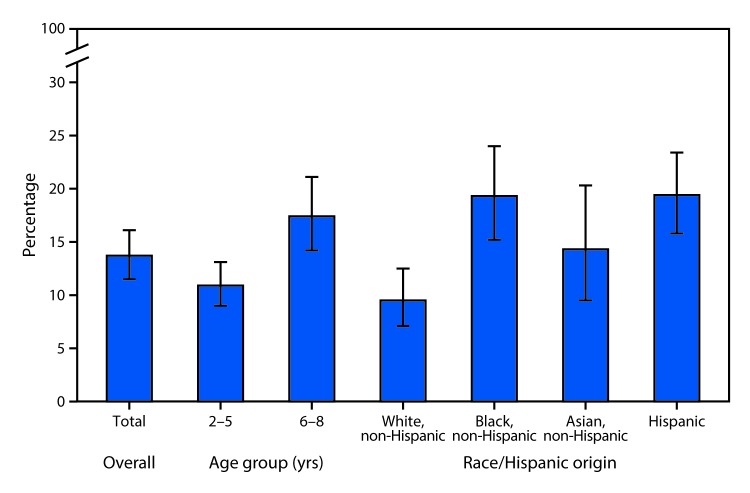
During 2011–2014, 13.7% of children aged 2–8 years had untreated dental caries in their primary teeth (baby teeth). The proportion of children with untreated dental caries in their primary teeth increased with age: 10.9% among children aged 2–5 years and 17.4% among children aged 6–8 years. A larger proportion of Hispanic (19.4%) and non-Hispanic black children (19.3%) had untreated dental caries in primary teeth compared with non-Hispanic white (9.5%) children.

**Source:** CDC/NCHS. National Health and Nutrition Examination Survey Data. Hyattsville, MD: US Department of Health and Human Services, CDC, National Center for Health Statistics; 2011–2014. https://www.cdc.gov/nchs/nhanes.htm.

